# Supplementary material for: In-cell NMR suggests that DNA i-motif levels are strongly depleted in living human cells
Source: Nat Commun. 2024 Mar 5;15:1992. doi: 10.1038/s41467-024-46221-y (PMC10914786; doi:10.1038/s41467-024-46221-y)
Supplement: Supplementary file 1 — Supplementary Information [file 41467_2024_46221_MOESM1_ESM.pdf]

SUPPLEMENTARY INFORMATION

In-cell NMR suggests that DNA i-motif levels are strongly depleted in living human cells.

Pavlína Víšková[1,2 #], Eva Ištvánková[1,2 #], Jan Ryneš[1], Šimon Džatko[1,3], Tomáš Loja[1], Martina Lenarčič Živković[1,4], Riccardo Rigo[1,5], Roberto El-Khoury[6], Israel Serrano-Chacón[7], Masad J. Damha[6], Carlos González[7], Jean-Louis Mergny[8,9], Silvie Foldynová-Trantírková[1,8*] and Lukáš Trantírek[1*].

[1] Central European Institute of Technology, Masaryk University, 625 00 Brno, Czech Republic
[2] National Centre for Biomolecular Research, Masaryk University, 625 00 Brno, Czech Republic
[3] Centre for Advanced Materials Application, Slovak Academy of Sciences, 845 11 Bratislava, Slovakia
[4] Slovenian NMR Centre, National Institute of Chemistry, SI-1000 Ljubljana, Slovenia
[5] Dipartimento di Scienze del Farmaco, University of Padova, 5-Padova, Italy
[6] Department of Chemistry, McGill University, Montreal, QC, H3A0B8, Canada
[7] Instituto de Química Física 'Rocasolano', CSIC, C/Serrano 119, 28006 Madrid, Spain
[8] Institute of Biophysics, Czech Academy of Sciences, Brno, Czech Republic
[9] Laboratoire d'Optique & Biosciences, Institut Polytechnique de Paris, Inserm, CNRS, Ecole Polytechnique, Palaiseau 91120, France.

[#] Equally contributing authors
To whom correspondence should be addressed: silvie.trantirkova@ceitec.muni.cz, lukas.trantirek@ceitec.muni.cz.

Supplementary Table 1: List of DNA constructs used in this study.

Name	Sequence (5‘ → 3‘)
hybrid-ds/hT121-6 FW	GCT TCT AGT CAA TCC CCC CTC CCC CCT TCC CCC CTC CCC CC
FAM_ hybrid-ds/hT121-6 FW	[6FAM] GCT TCT AGT CAA TCC CCC CTC CCC CCT TCC CCC CTC CCC CC
hybrid-ds/hTel FW	GCT TCT AGT CAA T CCC TAA CCC TAA CCC TAA CCC
FAM_ hybrid-ds/hTel FW	[6FAM] GCT TCT AGT CAA T CCC TAA CCC TAA CCC TAA CCC
hybrid-ds/hPDGFa FW	GCT TCT AGT CAA TCC GCG CCC CTC CCC CGC CCC CGC CCC CGC CCC CCC CCC CCC
FAM_ hybrid-ds/hPDGFa FW	[6FAM] GCT TCT AGT CAA TCC GCG CCC CTC CCC CGC CCC CGC CCC CGC CCC CCC CCC CCC
hybrid-ds/hRAD17 FW	GCT TCT AGT CAA TCC ACC CCC CCC CGC CCC CCC CCG GA
FAM_hybrid-ds/hRAD17 FW	[6FAM] GCT TCT AGT CAA TCC ACC CCC CCC CGC CCC CCC CCG GA
hybrid-ds RV	TTG ACT AGA AGC
hybrid-ds FW	GCT TCT AGT CAA
hBcl-2	CAG CCC CGC TCC CGC CCC CTT CCT CCC GCG CCC GCC CCT
FAM_hBcl-2	[6FAM] CAG CCC CGC TCC CGC CCC CTT CCT CCC GCG CCC GCC CCT
Cy3_hT121-6	[Cyanine3] CCC CCC TCC CCC CTT CCC CCC TCC CCC C
Cy3_hTel	[Cyanine3] CCC TAA CCC TAA CCC TAA CCC
Cy3_hPDGFa	[Cyanine3] CCG CGC CCC TCC CCC GCC CCC GCC CCC GCC CCC CCC CCC CC
Cy3_hRAD17	[Cyanine3] CCA CCC CCC CCC GCC CCC CCC CGG A
Cy3_hBcl-2	[Cyanine3] CAG CCC CGC TCC CGC CCC CTT CCT CCC GCG CCC GCC CCT
Cy3_cMycG	[Cyanine3] TGG GGA GGG TGG GGA GGG TGG GGA AGG TGG GGA GAA GA
Cy3_hTelG	[Cyanine3] GGG TTA GGG TTA GGG TTA GGG

			iM / **hybrid-ds/iM**		
			**Tm (°C)**		
iM	**pH 7**	**pH 6.5**	**pH 6**	**pH 5.5**	**pH 5**
hTel	n.d. / **n.d.**	15.9 ± 0.5 / **13.9 ± 0.9**	26.5 ± 0.3 / **29.2 ± 0.3**	40.5 ± 0.5 / **42.2 ± 0.4**	51.3 ± 0.2 / **53.4 ± 0.2**
hT121-6	24.9 ± 0.5 / **28.0 ± 0.4**	36.6 ± 0.5 /**38.7 ± 0.5**	48.3 ± 0.2 / **49.7 ± 0.7**	63.6 ± 0.3 / **65.2 ± 0.8**	74.3 ± 0.7 / **77.9 ± 0.6**
hPDGFa	28.8 ± 1.2 / **27.6 ± 0.9**	37.7 ± 0.3 / **37.4 ± 0.3**	50.8 ± 0.5 / **49.8 ± 0.7**	65.7 ± 1.2 / **64.7 ± 0.8**	-
hRAD17	33.8 ± 0.5 / **34.9 ± 0.6**	45.1 ± 0.7 / **44.3 ± 1.2**	55.3 ± 1.0 / **54.2 ± 2.5**	-	-
hBcl-2	12.9 ± 0.5	26.5 ± 0.2	38.1 ± 0.2	52.2 ± 0.4	-

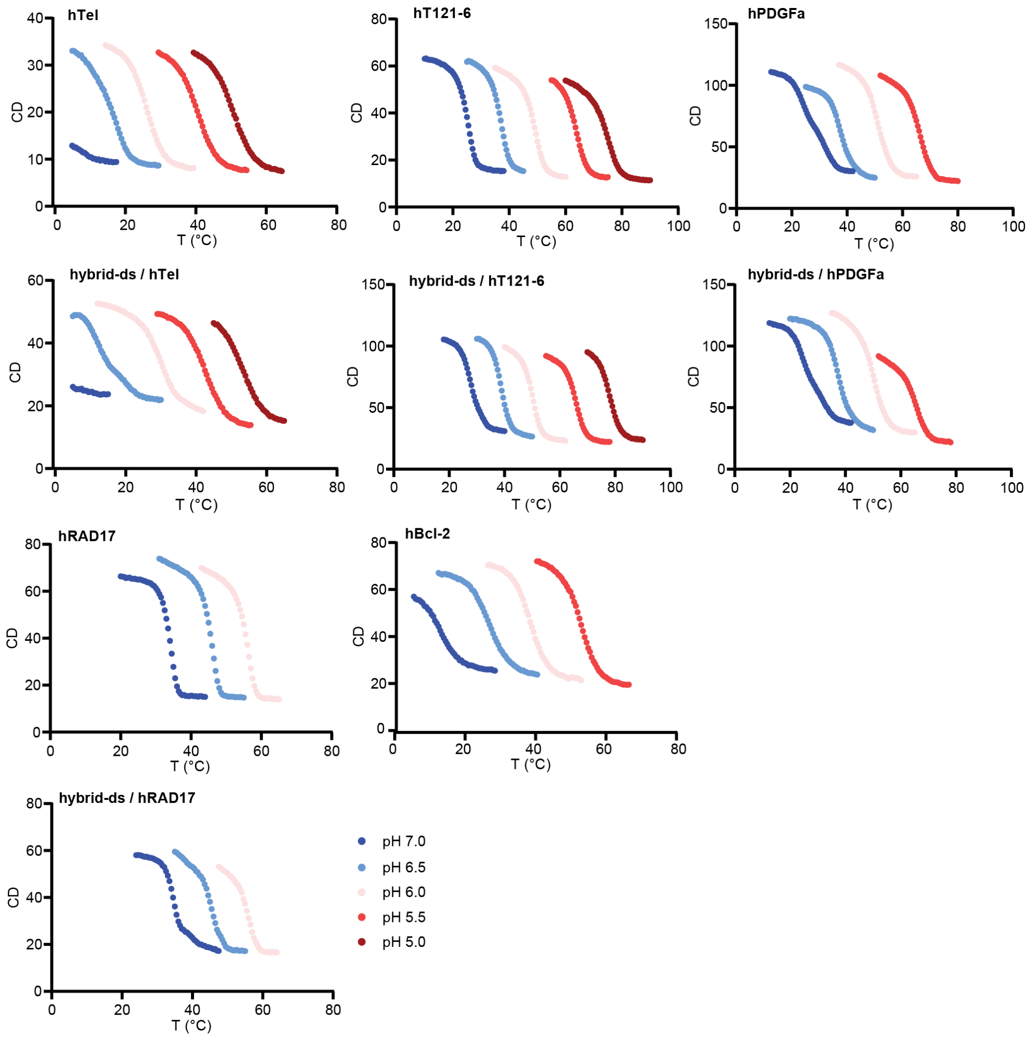

Supplementary Figure 1. Assessment of the thermal stability of iM and hybrid-ds/iM constructs at different pH in vitro. The $T_m^{in-vitro}$values were derived from CD melting profiles of iM and hybrid-ds/iM constructs measured at different pH (as indicated). Each sample was prepared in IC-cacodylate-based buffer (25 mM sodium cacodylate, 10.5 mM NaCl, 110 mM KCl, 1 mM $MgCl_2$, 130 nM $CaCl_2$). The reported error corresponds to the standard error of the mean for each triplicate experiment. Numbers in type set in regular and bold font correspond to iM and hybrid-ds/iM constructs, respectively.

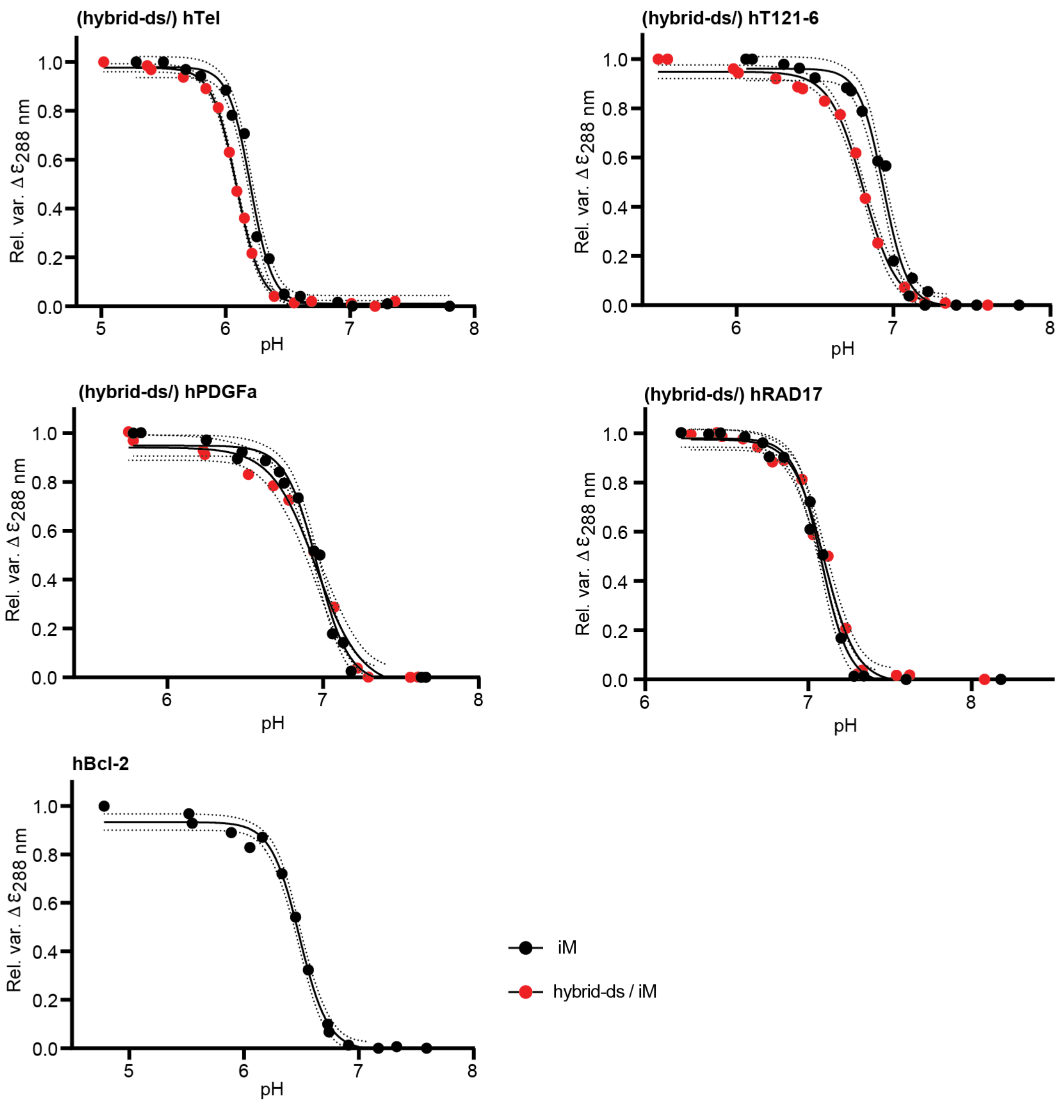

Supplementary Figure 2. Assessment of pH-dependency of iM and hybrid-ds/iM constructs. The graphs show the CD signal variations (monitored at 288 nm) as a function of the pH change. Black and red dots correspond to the pH titration for the iM and hybrid-ds/iM, respectively. The measurements were performed at 20 °C in IC buffer (25 mM potassium phosphate, 10.5 mM NaCl, 110 mM KCl, 1 mM $MgCl_2$, 130 nM $CaCl_2$). The panels represent the fitted curves as solid lines, while dotted lines indicate the 95% confidence interval of the fit. The derived $pH_T^{in-vitro}$ values are reported in Table 1.

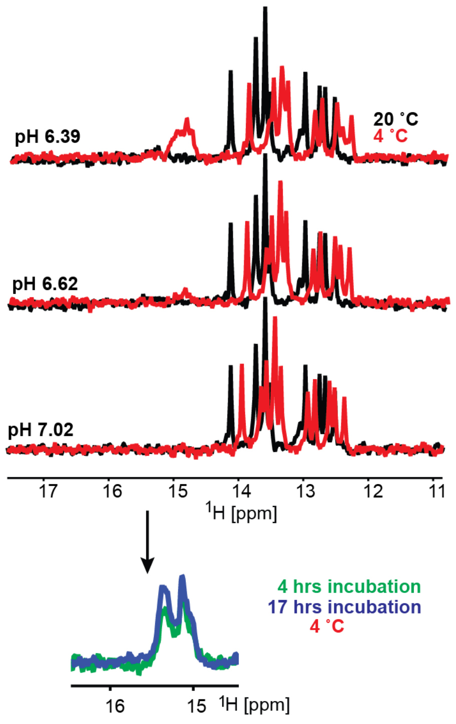

Supplementary Figure 3. iM formation in hybrid-ds/hTel depends on pH, temperature, and time. Overlays of the imino regions of 1D ^{1}H NMR spectra of hybrid-ds/hTel acquired in vitro (IC buffer: 25 mM KPO_i, 10.5 mM NaCl, 110 mM KCl, 1 mM $MgCl_2$, 130 nM $CaCl_2$) at 4 °C (red) and 20 °C (black), as a function of pH and the time at 4 °C (green and blue). Note: The temperature-induced chemical shift changes observed for Watson-Crick signals (12-14,5 ppm) are results of (de)stabilization of the double-helical region by temperature alteration (dynamical averaging).

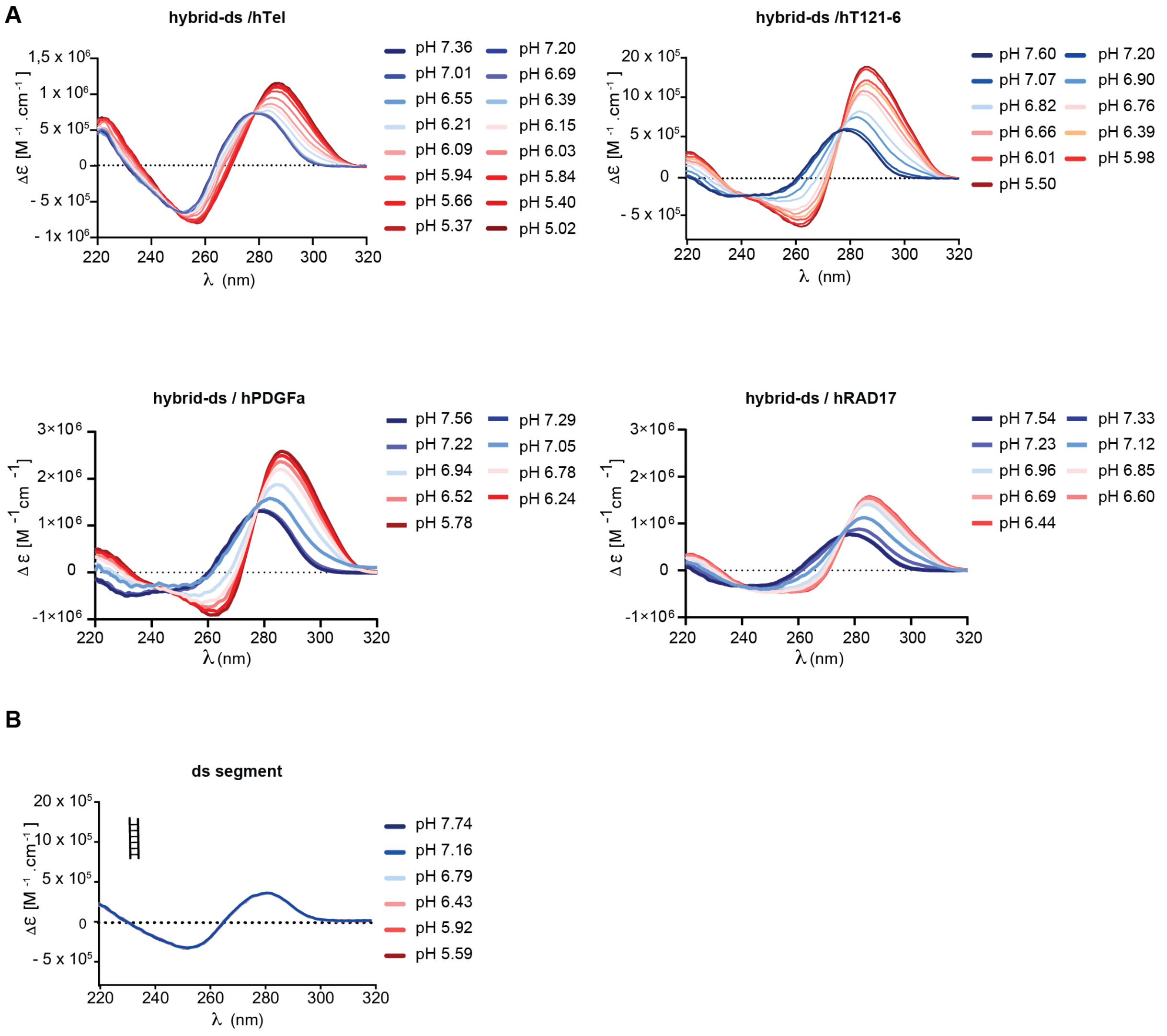

Supplementary Figure 4. **ds-segment does not impair iM folding within hybrid-ds/iM**. CD spectra of hybrid-ds/iM (A) and isolated ds segment (B) acquired as a function of pH at room temperature in vitro (IC buffer: 25 mM KPO_i, 10.5 mM NaCl, 110 mM KCl, 1 mM $MgCl_2$, 130 nM $CaCl_2$).

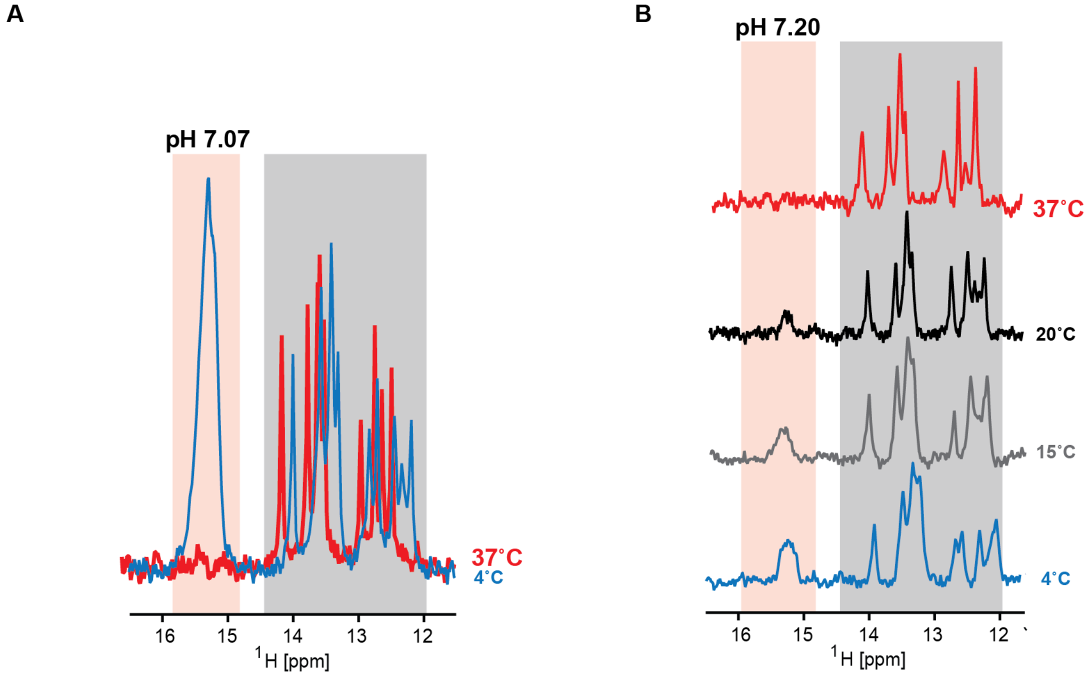

Supplementary Figure 5. Low temperature promotes iM folding within hybrid-ds/hT121-6. Imino regions of the 1D ^{1}H NMR spectra of hybrid-ds/hT121-6 acquired as a function of the temperature (indicated) in (A) IC buffer and (B) crude (buffered) cellular homogenate from HeLa cells. Red and gray boxes highlight imino signals specific to iM- and dsDNA-segments, respectively. Note that while the signal intensity pertinent to iM (highlighted in the red box) observed at 4 °C was essentially reduced to zero when the temperature was increased to 37 °C, the alterations in the NMR signal intensities (integral intensities – considering temperature-dependent line broadening and the inherently low signal-to-noise ratio in the in-cell NMR spectra) corresponding to the dsDNA segment (gray box) were minimal.

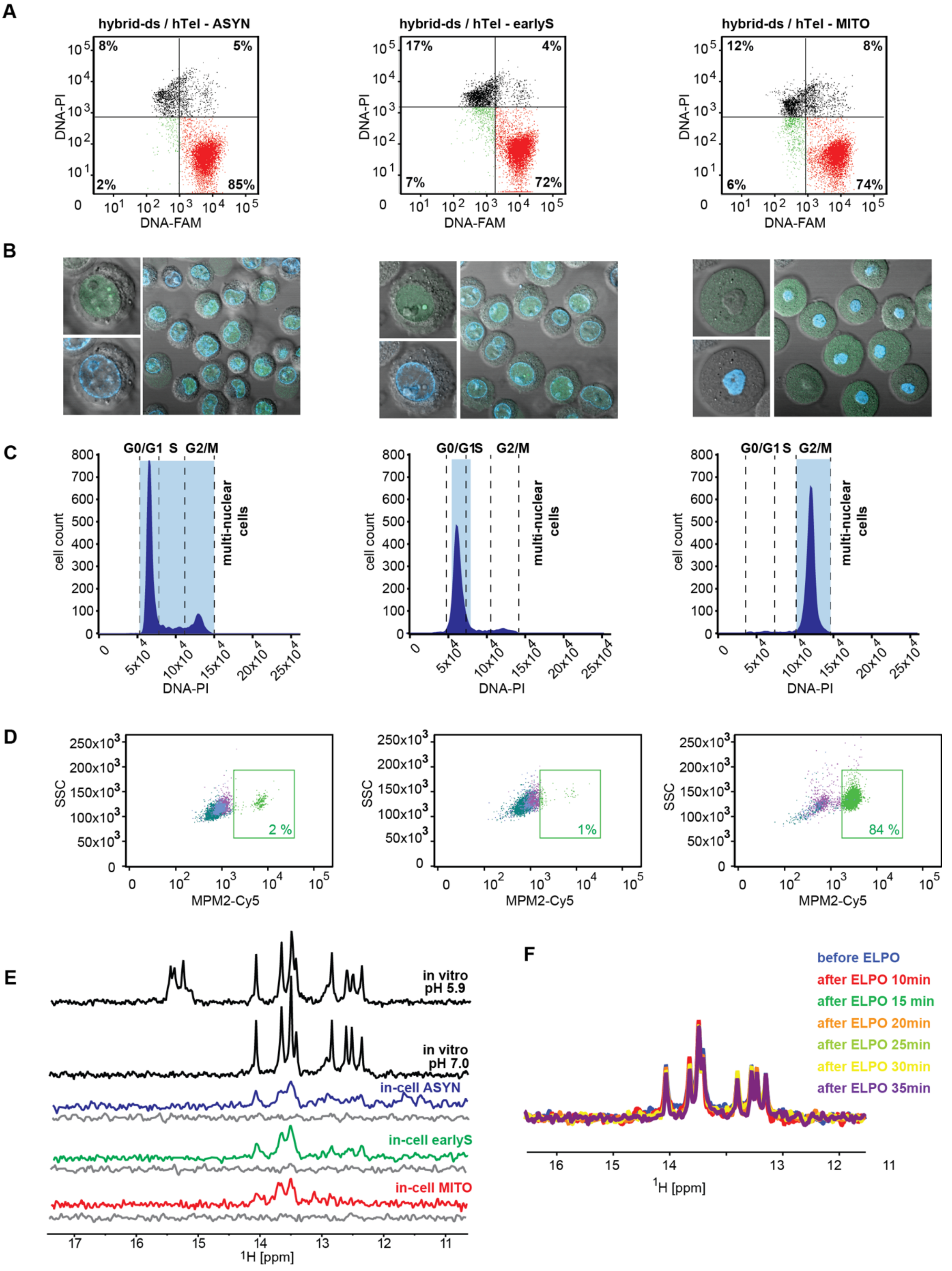

Supplementary Figure 6. **Controls for the in-cell NMR experiment with hybrid-ds/hTel**. (A) Double-staining (DNA-PI/DNA-FAM) FCM analysis (post-in-cell NMR acquisition) of asynchronous HeLa cells (left), HeLa cells synchronized in early-S (middle) and M- (right) cell-cycle phases transfected with (FAM-)hybrid-ds/hTel. The FCM plots indicate the

percentages of viable nontransfected cells: viable hybrid-ds/iM DNA-containing cells, dead/compromised nontransfected cells, and dead/compromised cells transfected with hybrid-ds/iMs in the bottom-left, bottom-right, top-left, and top-right quadrants, respectively. (B) and (C) show confocal microscopy images and propidium iodide (PI) DNA content staining of asynchronous HeLa cells (left), HeLa cells synchronized in early-S (middle), and M- (right) cells transfected with (FAM-)hybrid-ds/hTel. Green and blue colors in (B) indicate the localization of the introduced (FAM-)hybrid-ds/hTel and Hoechst-stained genomic DNA, respectively. (D) MPM2-Cy5 staining of asynchronous HeLa cells (left), HeLa cells synchronized in early-S (middle) and M- (right) cells transfected with (FAM-)hybrid-ds/hTel. (E) shows imino regions of 1D ^{1}H NMR spectra of hybrid-ds/hTel acquired in IC buffer (black), and in asyn- (blue), early-S (green) and M-(red) synchronized cells at 20 °C. The corresponding region of the 1D ^{1}H NMR spectra of the extracellular fluid taken from the samples after in-cell NMR spectra acquisition is shown in gray (leakage control). (F) show an overlay of imino regions of 1D ^{1}H NMR spectra of hybrid-ds/hTel before and after applying the ”mock” electroporation pulse.

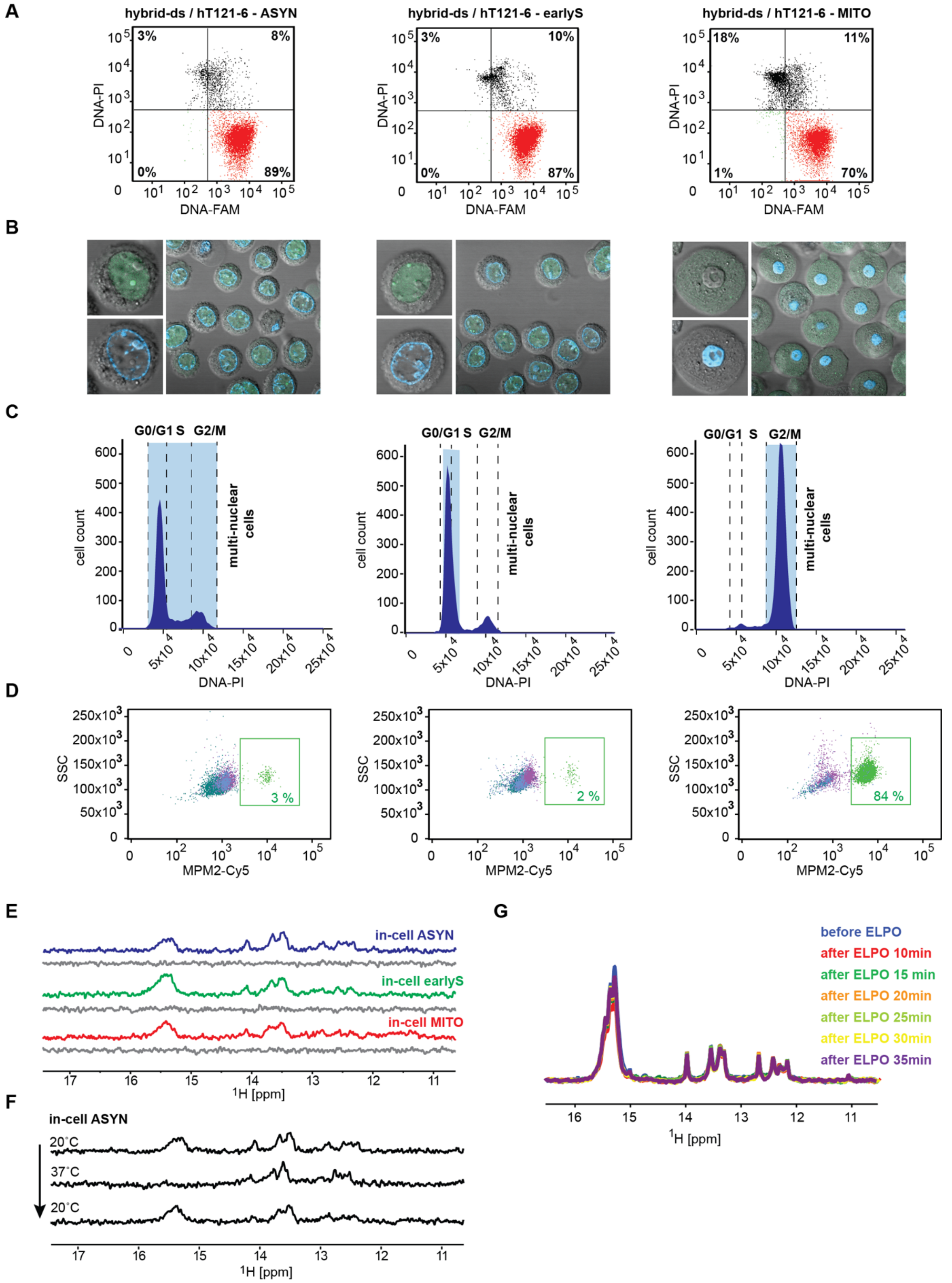

Supplementary Figure 7. **Controls for the in-cell NMR experiment with hybrid-ds/hT121-6.** (A) Double-staining (DNA-PI/DNA-FAM) FCM analysis (post-in-cell NMR acquisition) of asynchronous HeLa cells (left), HeLa cells synchronized in early-S (middle) and M- (right) cell-cycle phases transfected with (FAM-)hybrid-ds/hT121-6. The FCM plots indicate the

percentages of viable nontransfected cells: viable hybrid-ds/iM DNA-containing cells, dead/compromised nontransfected cells, and dead/compromised cells transfected with hybrid-ds/iMs in the bottom-left, bottom-right, top-left, and top-right quadrants, respectively. (B) and (C) show confocal microscopy images and propidium iodide (PI) DNA content staining of asynchronous HeLa cells (left), HeLa cells synchronized in early-S (middle), and M- (right) cells transfected with (FAM-)hybrid-ds/hT121-6. Green and blue colors in (B) indicate the localization of the introduced (FAM-)hybrid-ds/hT121-6 and Hoechst-stained genomic DNA, respectively. (D) MPM2-Cy5 staining of asynchronous HeLa cells (left), HeLa cells synchronized in early-S (middle) and M- (right) cells transfected with (FAM-)hybrid-ds/hT121-6. (E) shows imino regions of 1D ^{1}H NMR spectra of hybrid-ds/hT121-6 acquired in IC buffer (black), and in asyn- (blue), early-S (green) and M-(red) synchronized cells at 20 °C. The corresponding region of the 1D ^{1}H NMR spectra of the extracellular fluid taken from the samples after in-cell NMR spectra acquisition is shown in gray (leakage control). (F) shows imino regions of 1D ^{1}H NMR spectra of hybrid-ds/hT121-6 acquired as a function of the temperature (indicated) in suspension of asynchronous HeLa cells. (G) shows an overlay of imino regions of 1D ^{1}H NMR spectra of hybrid-ds/hTel and hybrid-ds/hT121-6 before and after applying the ”mock” electroporation pulse.

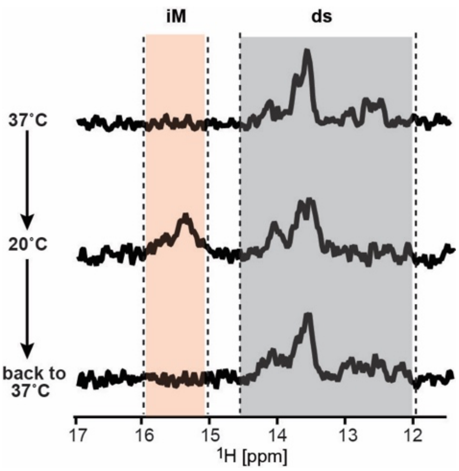

Supplementary Figure 8. **In-cell NMR experiment with hybrid-ds/hT121-6 using bioreactor**. Imino regions of the 1D ^{1}H NMR spectra of hybrid-ds/hT121-6 acquired as a function of the temperature (indicated) in low-melting agarose immobilized asynchronous HeLa cells under conditions of the constant media flow (bioreactor) with the acquisition time window of 30 min.

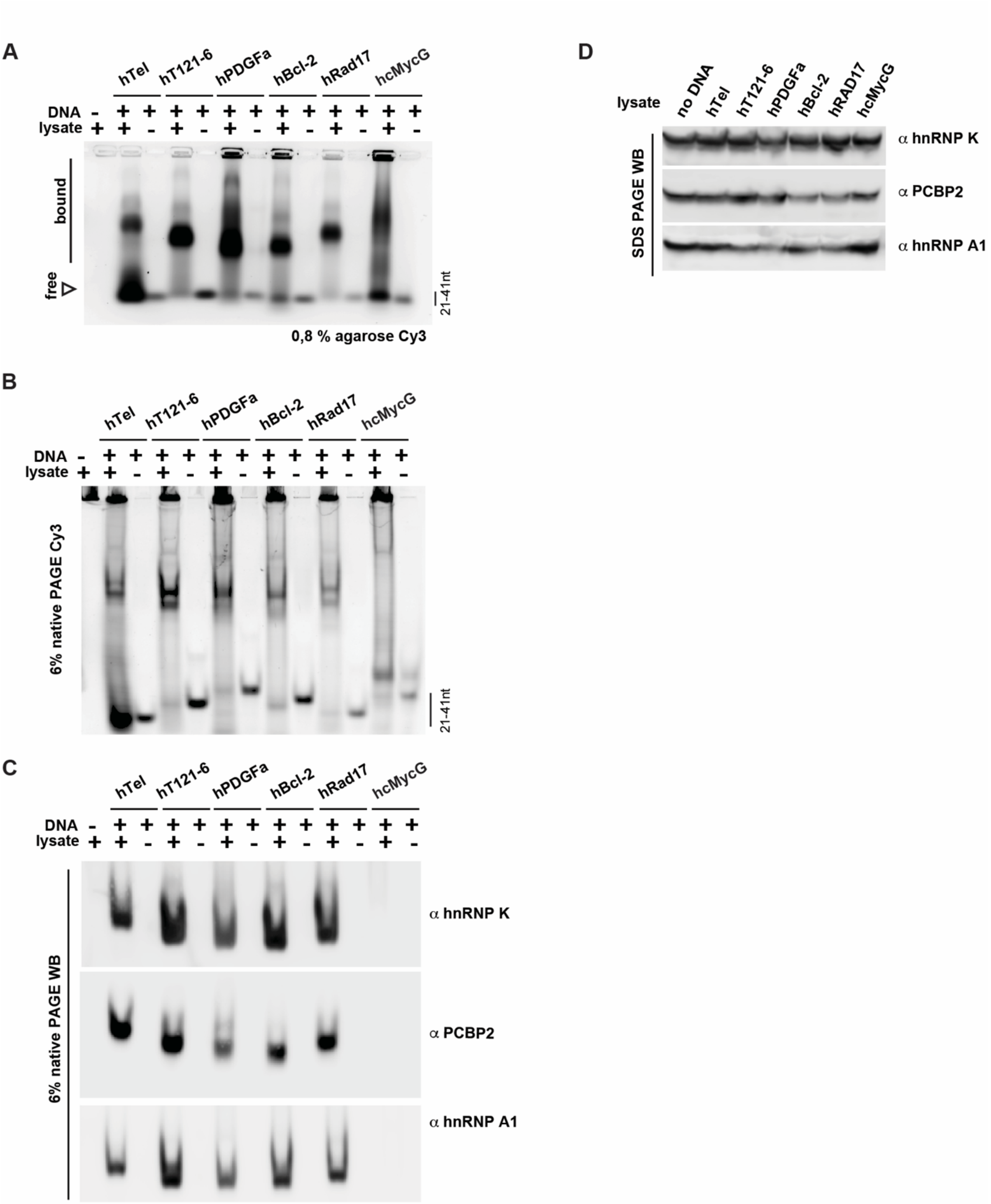

Supplementary Figure 9. Protein binding to DNA oligonucleotides. (A) Electrophoretic migration of the indicated Cy3-labelled oligonucleotides in agarose gel, in the absence and the presence of lysates from HeLa cells, visualized via the Cy3-fluorescence. (B) Native PAGE of the indicated Cy3-labelled oligonucleotides in the absence and the presence of lysates from HeLa cells, visualized via the Cy3-fluorescent label (6% native PAGE Cy3). (C) 6% native PAGE WB shows immunostaining of proteins transferred to the PVDF membrane from the native PAGE gel, using α hnRNP K, α PCBP2, and α hnRNP A1 antibodies. (D) Loading control showing the initial amounts of proteins in the HeLa cell lysates, detected with SDS-PAGE and Western blotting.

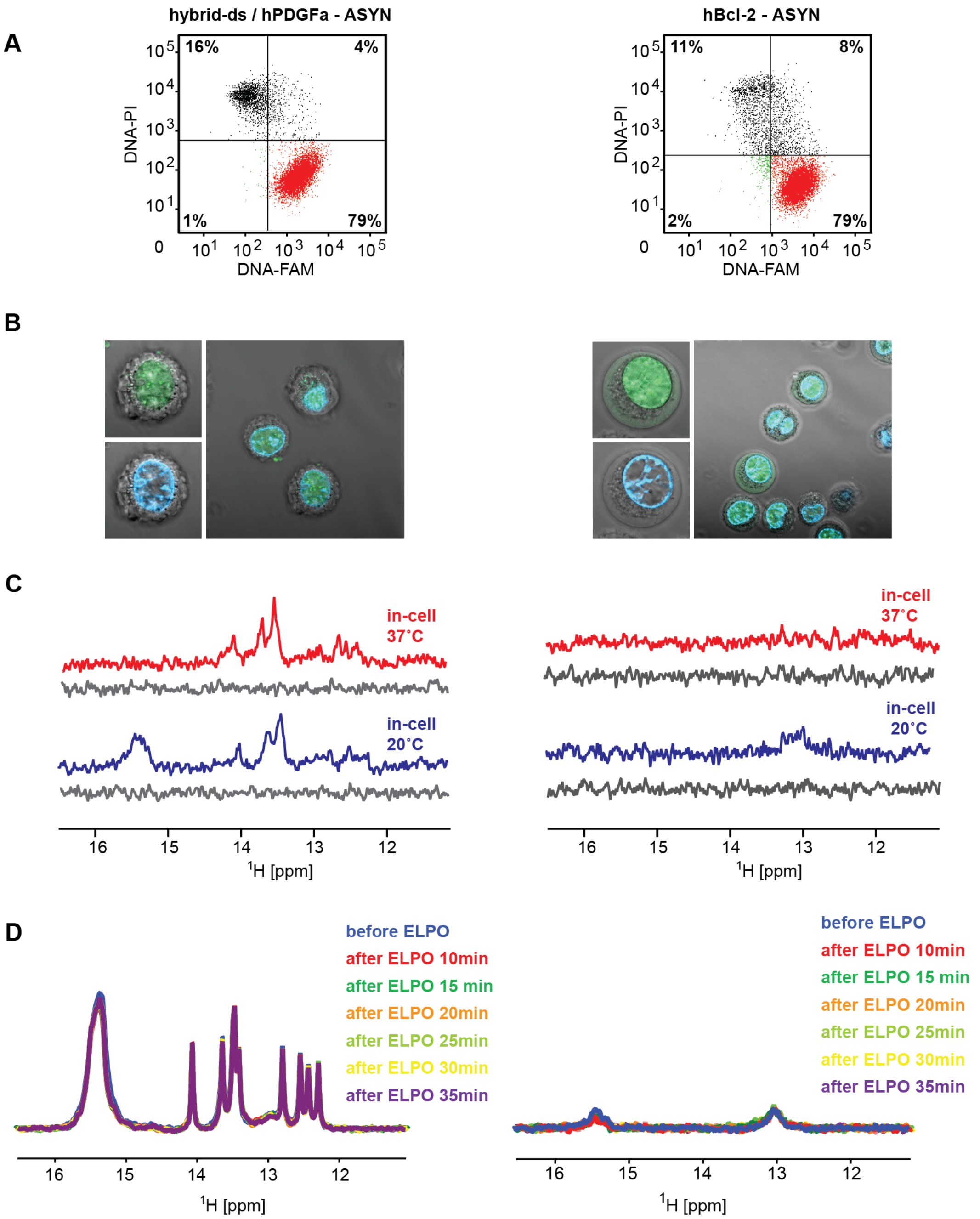

Supplementary Figure 10. Controls for the in-cell NMR experiment with (FAM-)hybrid-ds/hPDGFa and (FAM-)hBcl-2. (A) Double-staining (DNA-PI/DNA-FAM) FCM analysis of asynchronous HeLa cells transfected with (FAM-)hybrid-ds/hPDGFa (left) and (FAM-)hBcl-2 (right). The FCM plots indicate the percentages of viable nontransfected cells: viable DNA-containing cells, dead/compromised nontransfected cells, and dead/compromised transfected cells in the bottom-left, bottom-right, top-left, and top-right quadrants, respectively. (B) shows confocal microscopy images of asynchronous HeLa cells transfected with (FAM-)hybrid-ds/hPDGFa (left) and (FAM-)hBcl-2 (right). Green and blue indicate the localization of introduced FAM-labeled DNA and Hoechst-stained genomic DNA, respectively. (C) The imino regions of 1D ^{1}H in-cell NMR spectra of hybrid-ds/hPDGFa (left) and hBcl-2 (right) were acquired at 37 and 20 °C in asynchronous cells. The corresponding regions of 1D ^{1}H in

vitro NMR spectra of the extracellular fluid taken from the samples after in-cell NMR spectra acquisition are shown in gray (leakage control). (D) shows an overlay of imino regions of 1D ^{1}H in vitro NMR spectra of hybrid-ds/hPDGFa (left) and hBcl-2 (right) before and after applying the ”mock” electroporation pulse.

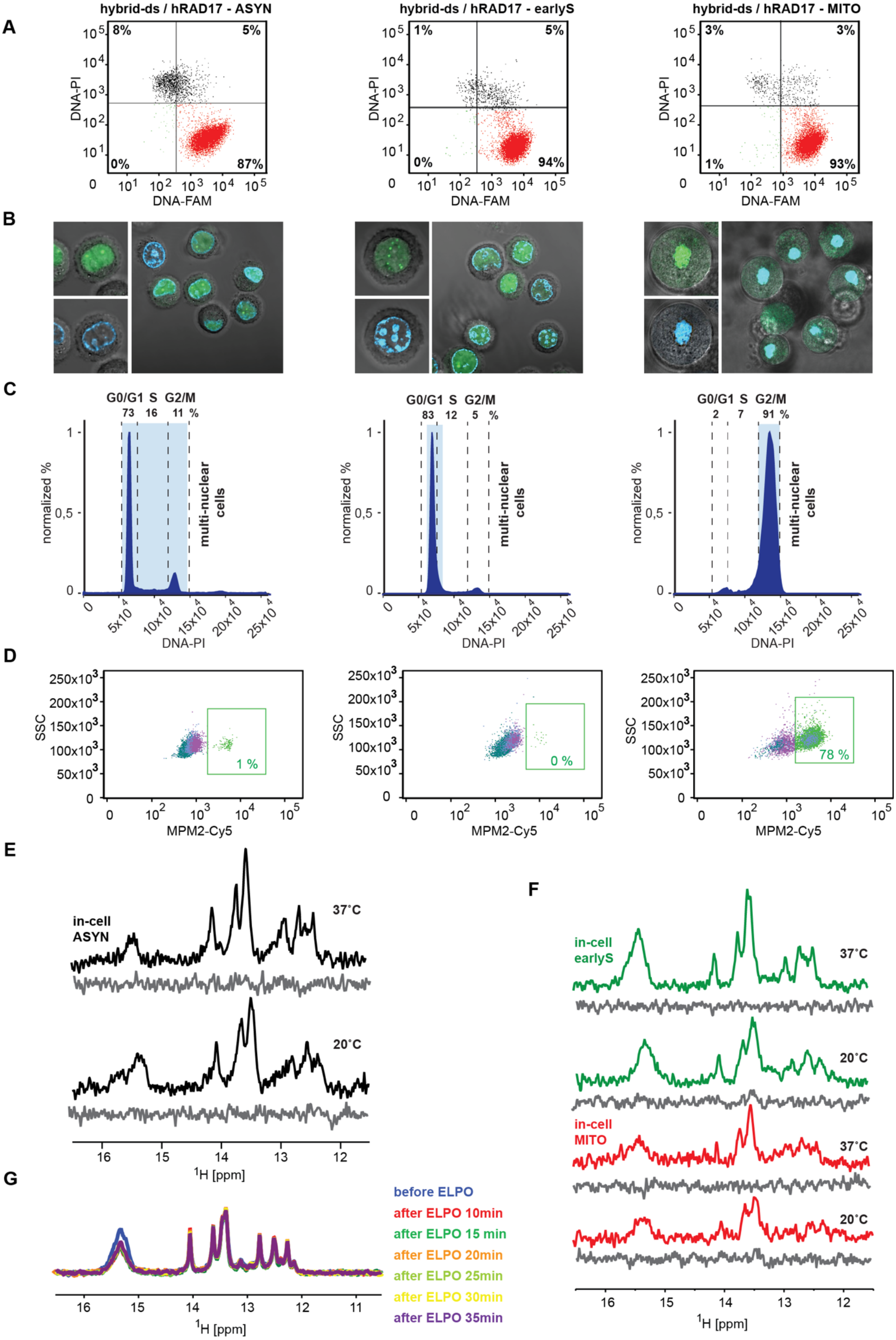

A
hybrid-ds / hRAD17 - ASYN
hybrid-ds / hRAD17 - earlyS
hybrid-ds / hRAD17 - MITO
DNA-PI
DNA-FAM
8%
5%
0%
87%
1%
5%
0%
94%
3%
3%
1%
93%
B
C
G0/G1 S G2/M
73 16 11 %
83 12 5 %
2 7 91 %
normalized %
multi-nuclear cells
DNA-PI
D
SSC
MPM2-Cy5
1 %
0 %
78 %
E
in-cell ASYN
37°C
20°C
^{1}H [ppm]
F
in-cell earlyS
in-cell MITO
37°C
20°C
^{1}H [ppm]
G
before ELPO
after ELPO 10min
after ELPO 15 min
after ELPO 20min
after ELPO 25min
after ELPO 30min
after ELPO 35min
^{1}H [ppm]

Supplementary Figure 11. Controls for the in-cell NMR experiment with (FAM-)hybrid-ds/hRAD17. (A) Double-staining (DNA-PI/DNA-FAM) FCM analysis and (B) confocal microscopy images of asynchronous (left), early-S (middle) and M-synchronized (right) HeLa cells transfected with (FAM-)hybrid-ds/hRAD17. Green and blue colors indicate the localization of FAM-labeled DNA and Hoechst-stained nuclear DNA, respectively. Synchronization controls: (C) Propidium iodide (PI) stained DNA content and (D) MPM2-Cy5 stained asynchronous (left), early-S (middle) and M-synchronized (right) HeLa cells transfected with (FAM-)hybrid-ds/hRAD17. The number in (D) indicates the percentages of mitotic cells in the population. (E) The imino regions of 1D ^{1}H in-cell NMR spectra of hybrid-ds/hRAD17 acquired at 37 and 20 °C in asynchronous cells and (F) early-S and M-synchronized Hela cells. The corresponding regions of 1D ^{1}H in vitro NMR spectra of the extracellular fluid taken from the samples after in-cell NMR spectra acquisition are shown in gray (leakage control). (G) shows an overlay of imino regions of 1D ^{1}H in vitro NMR spectra of hybrid-ds/hRAD17 before and after applying the ”mock” electroporation pulse.

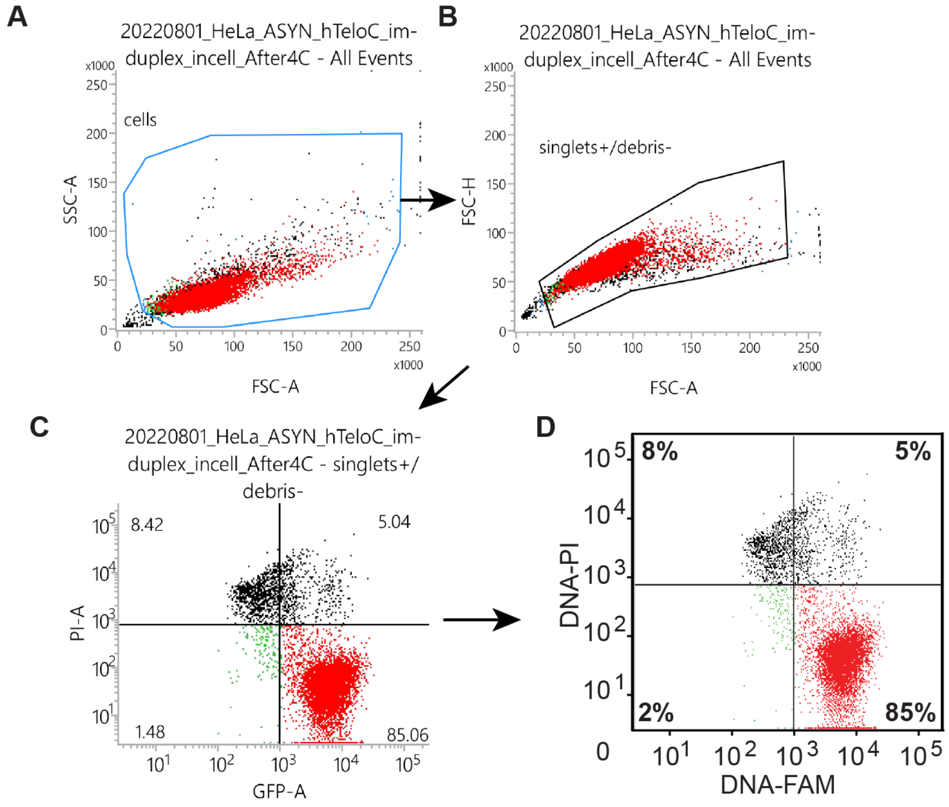

Supplementary Figure 12. An example of an FCS gating strategy employed to distinguish the apoptotic, dead cells or cells with compromised membrane integrity demonstrated on the FAM-hybrid-ds/hTel oligonucleotide transfected into asynchronous cells. (A) FSC-A and SSC-A gated cells to exclude the debris, (B) FSC-A/FSC-H gating to select singlet cells, (C) the final gating to visualize the cell viability (DNA-PI; y-axis) and the cell transfection efficiency (DNA-FAM; x-axis), and (D) the final processing by Adobe Illustrator CS6 V16.0.0.

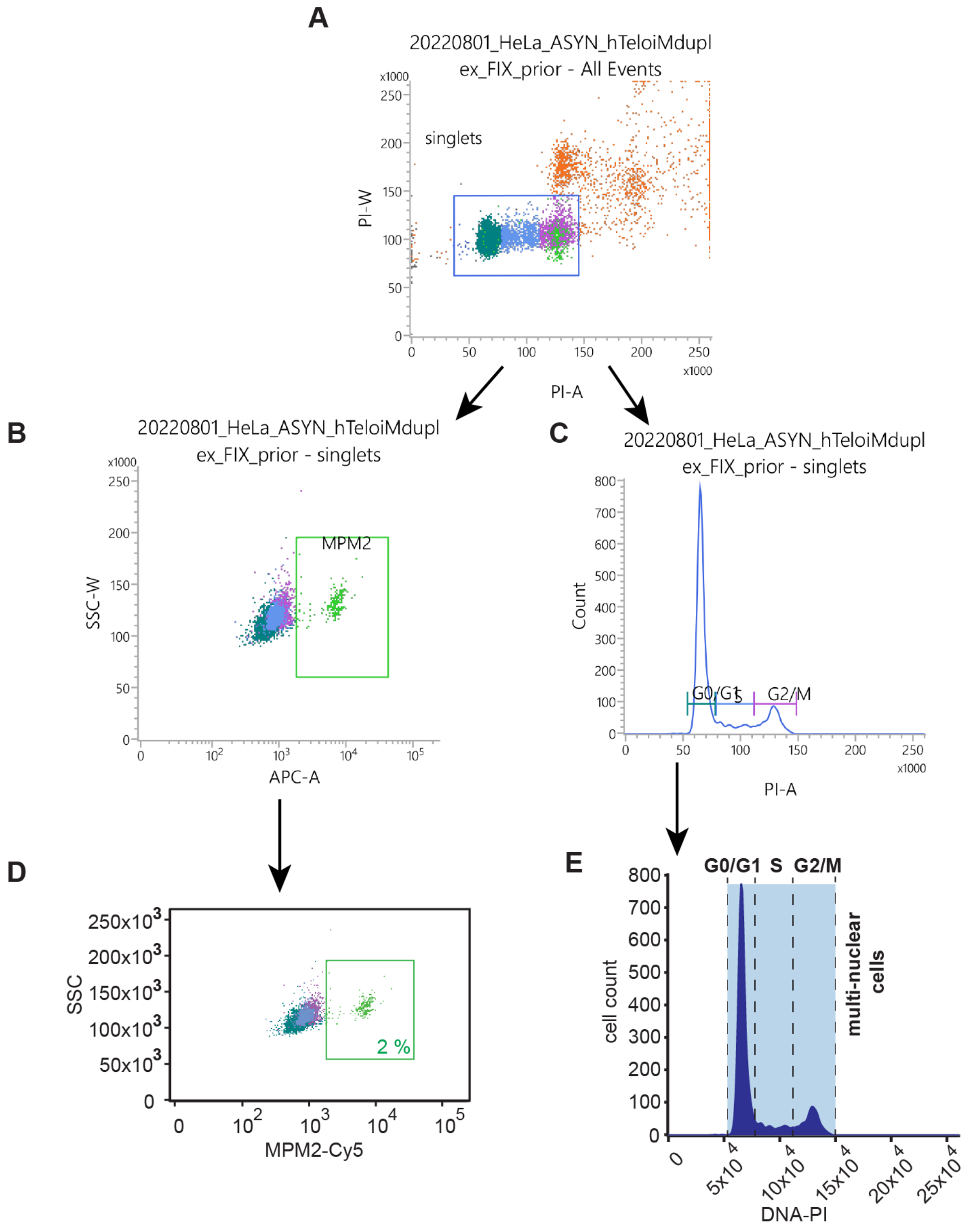

Supplementary Figure 13. An example of an FCS gating strategy to evaluate cell cycle synchronization demonstrated on the hybrid-ds/hTel oligonucleotide transfected into asynchronous cells. (A) PI-A/PI-W gating to select singlet cells, (B) final gating to visualize the population of mitotic cells (MPM2-Cy5; x-axis), (C) final gating to distinguish individual phases of the cell cycle (DNA-PI; x-axis). (D) and (E) show the final processing by Adobe Illustrator CS6 V16.0.0 of (B) and (C), respectively.

Supplementary Table 2: The potential technical issues impacting the in-cell NMR data interpretations regarding iM formation in-cells.

	**Commentary**
Perturbed metabolic homeostasis of the cells resulting from the overrepresentation of model iMFPS.	Overrepresenting the DNA fragment in the intracellular space might alter the native cellular environment, particularly the molecular crowding (MC) levels in the intracellular space: MC is known to promote iM formation under *in-vitro* conditions. Admittedly, the in-cell NMR readout can be biased towards iM formation. Nonetheless, the fact that in-cell NMR observed iM equilibria resemble those observed in diluted solutions at neutral pH *in-vitro* suggests that potential bias stemming from artificially increased MC is small. Note: NMR detected (freely tumbling) exogenous DNA fragments' intracellular concentrations are in the range 10-20 μM. Total (freely tumbling + bound) exogenous DNA fragments' intracellular concentrations are estimated in the range 70-90 μM.
Perturbed metabolic homeostasis of the cells resulting from the data acquisition in transfected pelleted cells.	Pelleting cells might induce hypoxia connected with acidification of the intracellular space. To minimize the impact of the metabolic changes, which might occur on a time scale longer than 30 minutes,[38,39] we deliberately shortened the NMR acquisition window to about 10 min. Admittedly, our readout could still be biased towards iM formation. Nonetheless, the fact that we observed an equilibrium shifts in the opposite direction (iM unfolding) either suggests that hypoxia-induced artifacts are limited in the given acquisition time or that even hypoxia-induced acidification cannot override the main regulatory factor (the temperature) repressing iM formation in human cells. This explanation aligns with the outcome of the control bioreactor-based experiment performed for hybrid-ds/hT121-6 with a prolonged acquisition time of about 30 minutes (Fig S5).
Restricted in-cell NMR data acquisition time.	Some previously studied iMFPS displayed slow iM folding kinetics compared to their unfolding. A question arises whether the used acquisition time of 10 minutes upon temperature increase to 37 ºC is enough for iMFPS to reach equilibrium. Admittedly, the readout could be biased towards iM unfolding. However, as demonstrated by the bioreactor-based in-cell NMR experiment for hybrid-ds/hT121-6, the respective iM remains unfolded in cells at 37 ºC even during three times longer acquisition window, 30 minutes (cf. Fig. S5). Both acquisition times are well above the timescale of processes related to the postulated iM roles, transcriptional regulators, for instance. Therefore, to affect the binding of transcription factors to their consensus sequences or to stop/activate the transcription, an iM in the gene promoter should/would have to fold in a timescale close to the activity of the transcriptional machinery: Transcription factors bind their specific DNA sites on a time scale of seconds and their residence time varies from seconds to less than 10 minutes.[64,65]

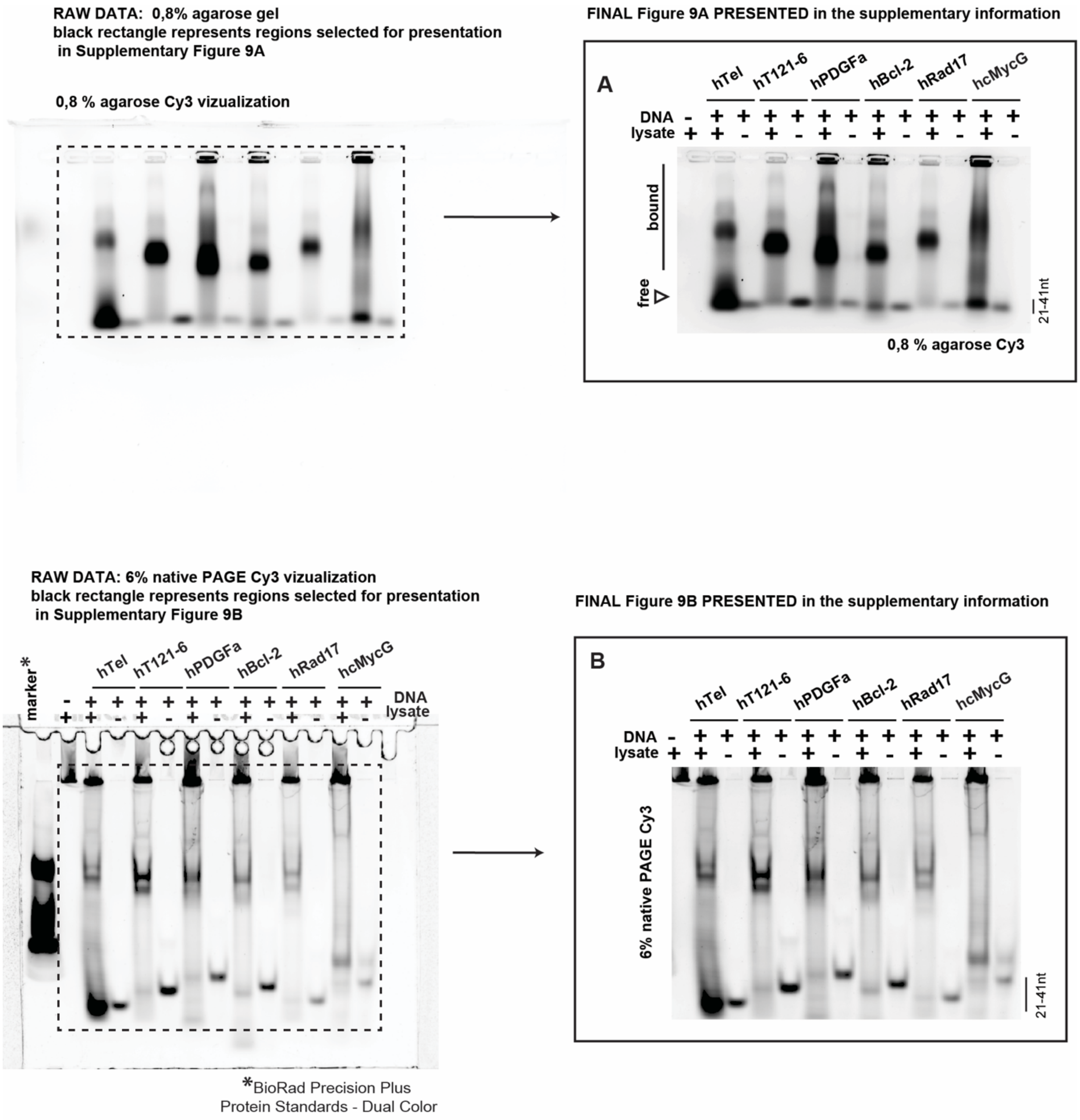

Supplementary Figure 14. Source Data for Supplementary Figure 9A (top) **and 9B** (bottom) - uncropped scans of blots and gels.

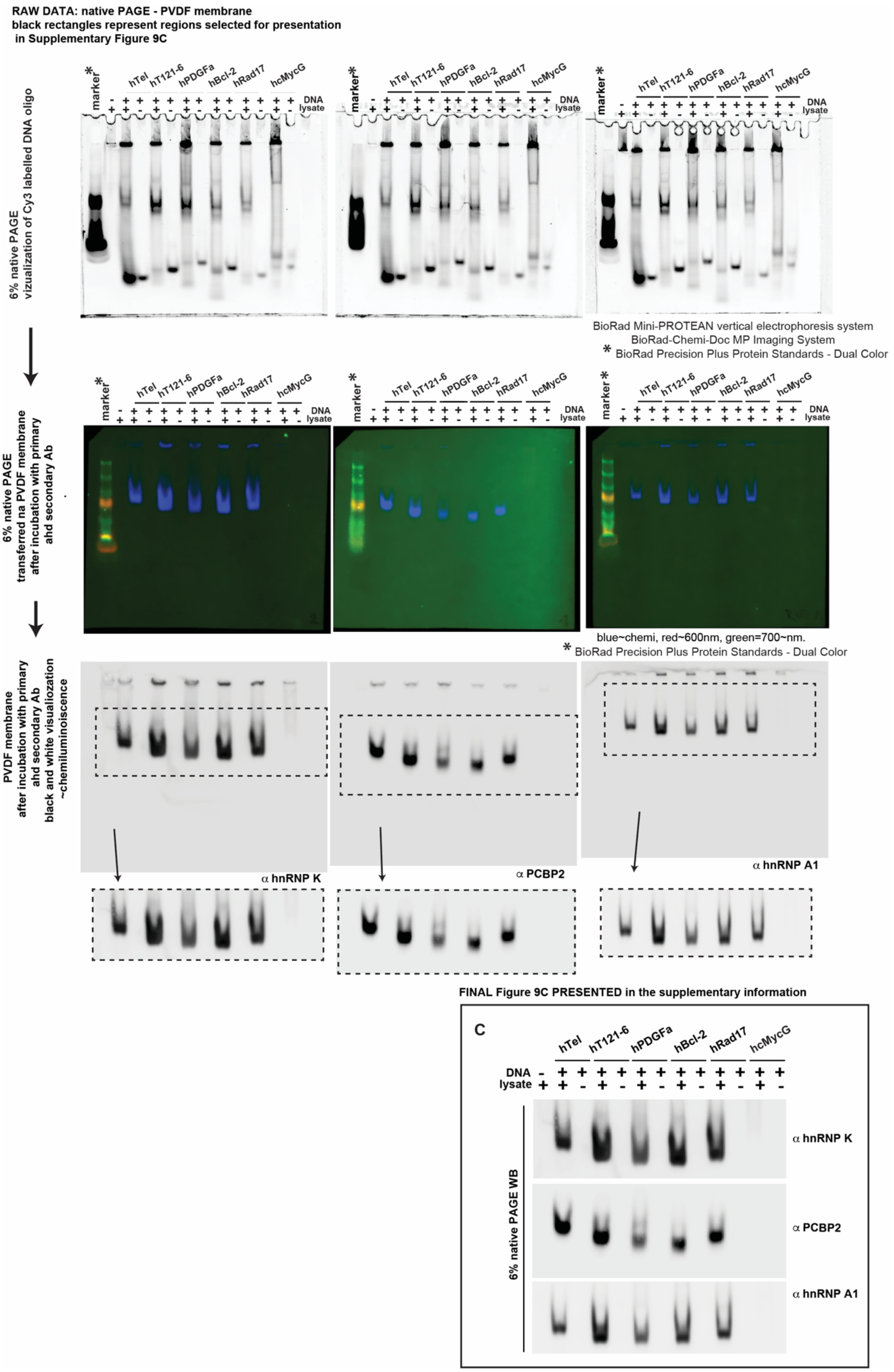

Supplementary Figure 15. Source Data for Supplementary Figure 9C - uncropped scans of blots and gels.

RAW DATA: PVDF membrane - WB
black rectangles represent regions selected for presentation
in Supplementary Figure 9D

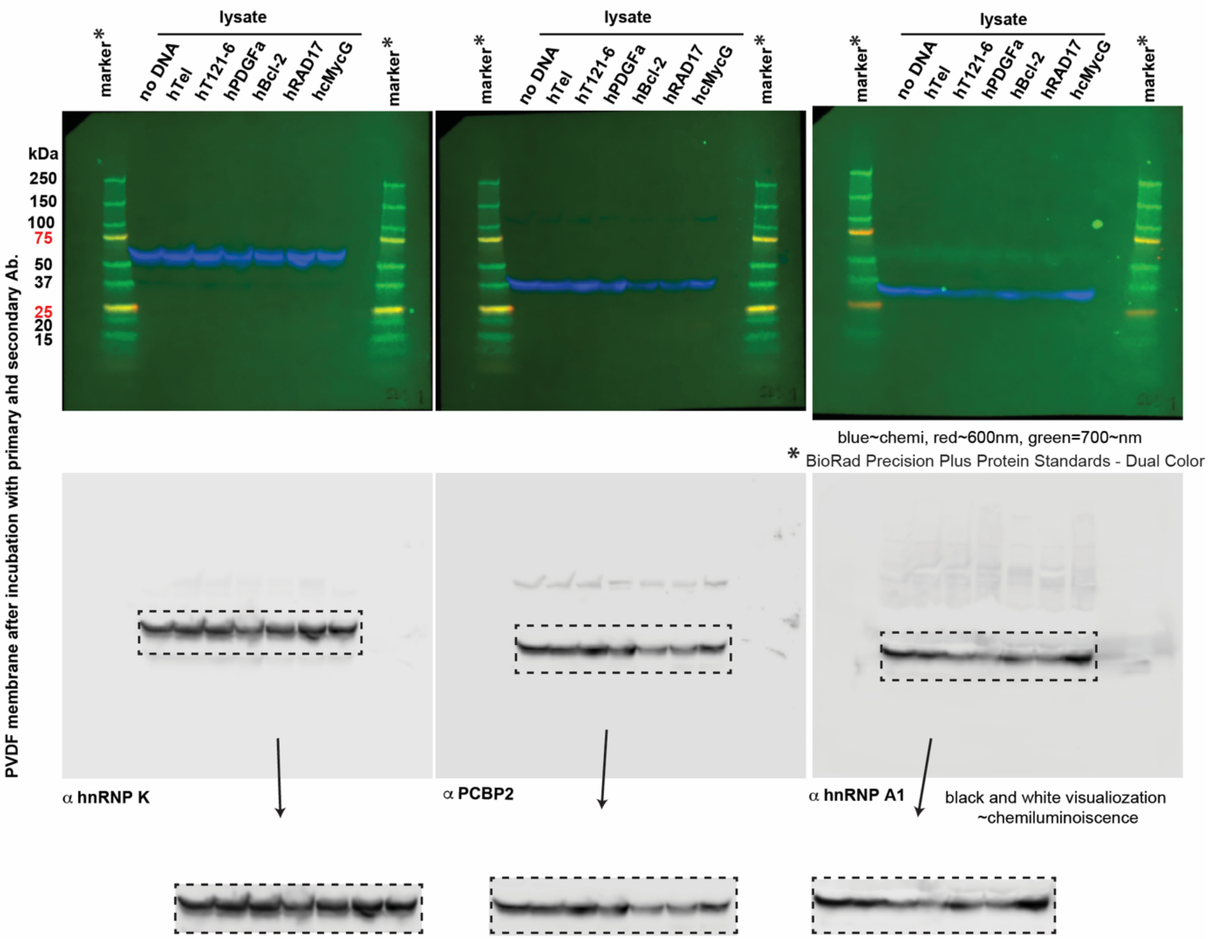

FINAL Figure 9D PRESENTED in the supplementary information

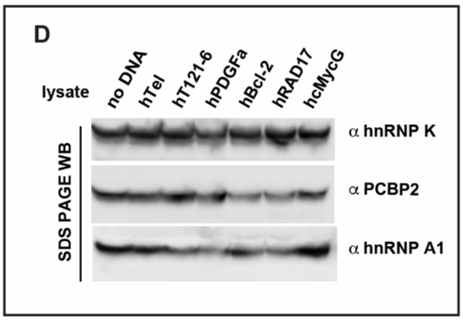

Supplementary Figure 16. Source Data for Supplementary Figure 9D - uncropped scans of blots and gels.

References:

64. Karpova, T. S. et al. Concurrent fast and slow cycling of a transcriptional activator at an endogenous promoter. *Science* **319**, 466–9 (2008).

65. Morisaki, T., Müllerm, W. G., Golob, N.,Mazza, D. & McNally, J. G. Single-molecule analysis of transcription factor binding at transcription sites in live cells. *Nat. Commun*. **5**, 4456 (2014).
